# Enhancement of Broadband Reverse Saturable Absorption of Red/Black Phosphorus Heterojunction

**DOI:** 10.3390/molecules29061271

**Published:** 2024-03-13

**Authors:** Jingjing Wang, Fulai Liu, Yunfei Li, Long Chen, Yong Chen, Hailong Zhang, Zheng Xie

**Affiliations:** 1 State Key Laboratory for Advanced Metals and Materials, University of Science and Technology Beijing, Beijing 100083, China; 2 Key Laboratory of Photochemical Conversion and Optoelectronic Materials, Technical Institute of Physics and Chemistry, Chinese Academy of Sciences, Beijing 100190, China

**Keywords:** red/black phosphorus, lateral heterojunction, reverse saturable absorption, optical limiting, nonlinear optics

## Abstract

Although laser technology brings convenience to production and daily life, it also causes high-energy damage. Therefore, there is an urgent need to develop optical limiting materials for laser protection. In this study, a novel nonlinear optical material, red/black phosphorus lateral heterojunction, is successfully prepared through solvothermal and ultrasonic treatment. Using the Z−scan method, the nonlinear optical properties of the red/black phosphorus heterojunction are determined at wavelengths of 532 and 1064 nm. These results indicate that the red/black phosphorus heterojunction exhibits reverse saturable absorption properties in 1.2.3-glycerol. Interestingly, the red/black phosphorus heterojunction shows an enhanced performance over red phosphorus by introducing the black phosphorus phase. Moreover, the red/black phosphorus heterojunction is doped into organically modified silicate gel glass with excellent broadband optical limiting performance. This study highlights the promising prospect of the red/black phosphorus heterojunction in the nonlinear optical and optical limiting fields.

## 1. Introduction

Since the invention of the first ruby laser in 1960, laser technology has made rapid progress [1]. In recent years, many laser technologies have emerged and brought great convenience to humanity, such as laser ranging [2], laser processing [3], laser display [4], and laser sensing [5]. However, high-energy damage to the human eyes or optical system also occurs at the same time [6,7]. For example, the laser radar used for intelligent assisted driving can cause luminous flux overloading in optical sensor devices, which consequently causes irreversible damage to the camera [8]. These types of laser damage seriously hinder production and human life, cause economic losses, or endanger physical health. The heavy demand for laser safety has rapidly driven the development of optical limiting (OL) technology [9]. OL based on third-order nonlinear optics (NLO) can effectively block high-energy light while allowing low-energy light to pass through, which can precisely address this issue. The transmittance of the medium decreases with the power density of incident light so that the laser energy can be limited to below the laser damage threshold [10]. As carriers, the NLO materials play an important role in achieving OL functionality [11]. There is a wide range of materials that can be used for optical limiting, such as organic polymers [12], inorganic metal compounds [13], and composites [14]. For several years, researchers have been attempting to explore new NLO materials with larger modulation depths, lower initial limiting thresholds, faster response times, and wider response wavebands. Usually, materials with multiple photon absorption [15,16], excited state absorption [17,18], and free carrier absorption [19,20] properties exhibit excellent OL performance. For example, Lim et al. observed that the excited state absorption of small π-electron systems plays an important role in the giant broadband nonlinear optical absorption of dispersed graphene single sheets [21]. In another study, He et al. reported the two-photon absorption processes of yttrium lithium fluoride doped thulium crystals and emphasized their potential application prospect in solid-state OL [22]. OL based on the excited state absorption mechanism requires NLO materials with stronger excited state absorptions, compared to ground state absorptions. The excited state absorption can be enhanced by increasing the excited state absorption cross-section and the population of excited state charge carriers. It is an effective method for realizing both the above aspects by forming heterojunctions to generate electron transfer.

Two-dimensional (2D) heterojunctions exhibit remarkable carrier transfer properties, making them excellent candidates for NLO [23,24], photocatalysis [25,26], and photodetection [27,28]. 2D heterojunctions can be divided into two types, lateral heterostructure and vertical heterostructure (stacked layer by layer) [29]. Lateral heterostructure has attracted much attention due to its lower charge delocalized barrier and better charge transport pathways. This material structure provides significant advantages for OL. On the one hand, the charge lifetime of intersystem crossing between phases in heterojunction is longer, compared with that of vibration relaxation, which can increase the population of excited state electrons. On the other hand, the absorption cross-section of the introduced second phase has a strong influence on the excited state absorption of the heterojunction. A larger absorption cross-section of the second phase results in a stronger OL performance [30]. Phosphorus-based materials are excellent choices for 2D heterojunctions used in OL due to their strong visible light absorptions and tunable bandwidths. Black phosphorus (BP) is known for its giant third-order NLO broadband response, serving as an excellent second phase of the heterojunction with strong absorption.

In this study, we successfully prepared a red/black phosphorus (RP/BP) lateral heterojunction by solvothermal and ultrasonic methods. During the solvothermal process, the phase transition was induced through ethylenediamine under high pressure. Under 532 and 1064 nm nanosecond laser excitation, the nonlinear optical performance of the RP/BP lateral heterojunction was characterized by a Z−scan system. The results indicate that the RP/BP heterojunction exhibits reverse saturable absorption characteristics in 1.2.3-glycerol (GI). Compared to red phosphorus (RP), the RP/BP heterojunction shows a significant enhancement in reverse saturable absorption due to the charge transfer. This material was doped into organically modified silicate (ormosil) gel glass as an OL device. The results indicate that the RP/BP heterojunction doped ormosil gel glass exhibits excellent reverse saturable absorption performance, applicable in the OL field.

## 2. Results and Discussion

**Figure 1:**
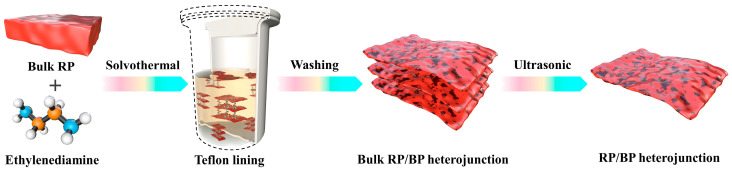
Schematic illustration of the preparation of the RP/BP heterojunction.

The RP/BP heterojunction is prepared by solvothermal and ultrasonic methods. As shown in Figure 1, the preparation process includes the phase transition of the bulk RP by solvothermal treatment, the purification process of the bulk RP/BP heterojunction by centrifugal treatment, and the exfoliation process of the bulk RP/BP heterojunction by ultrasonic treatment. Under high temperature and pressure conditions, bulk red phosphorus is intercalated and activated by ethylenediamine to produce phase transition [31]. Other solvents, such as water and ethanol, cannot induce phase transition of bulk red phosphorus under the same high temperature and pressure conditions [31]. The bulk RP/BP heterojunction obtained from solvothermal treatment is black in color. Through ultrasonic treatment, the bulk RP/BP heterojunction transforms into a layered-structured RP/BP heterojunction, and the color of the sample transforms into yellow.

**Figure 2:**
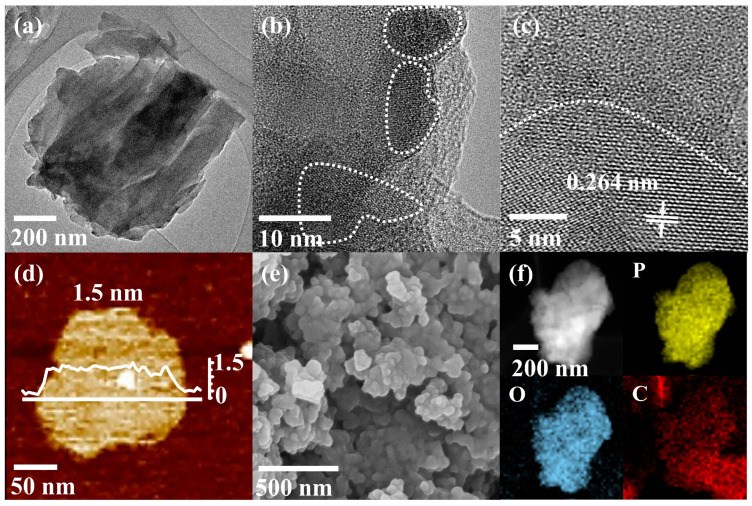
Morphology characterization of the RP/BP heterojunction. (**a**) TEM image; (**b**,**c**) HRTEM images; (**d**) AFM image; (**e**) SEM image; (**f**) EDS mappings of P, O, and C.

Transmission electron microscopy (TEM) is used to characterize the morphology and structure of the RP/BP heterojunction (Figure 2). The RP/BP heterojunction has a distinct sheet-like structure and an irregular edge shape (Figure 2a). The high-resolution TEM (HRTEM) image proves that this material belongs to a lateral heterostructure (Figure 2b). The partial areas of this material are crystalline phase (within the dashed circle in Figure 2b), while the remaining areas are amorphous phase (outside the dashed circle in Figure 2b). The spacing between the lattice fringe of the RP/BP heterojunction is 0.264 nm, corresponding to an (0 4 0) crystal plane of BP (Figure 2c) [32]. The thickness of the RP/BP heterojunction is ~1.5 nm, evidenced by an atomic force microscopy (AFM) image (Figure 2d). The scanning electron microscopy (SEM) image shows that the RP/BP heterojunction has a homogeneous morphology and a uniform size (Figure 2e). The RP/BP heterojunction is stacked together in layers. The energy dispersive spectrometer (EDS) mappings display the elemental composition of the RP/BP heterojunction (Figure 2f). The RP/BP heterojunction is composed of P, O, and C elements, among which the P element is dominant. The O element is related to the oxidation state of the RP/BP heterojunction at the structural edge and gas adsorption. Since the RP/BP heterojunction is attached to a carbon film for characterization, there is also a distribution of C elements around the RP/BP heterojunction sample.

**Figure 3:**
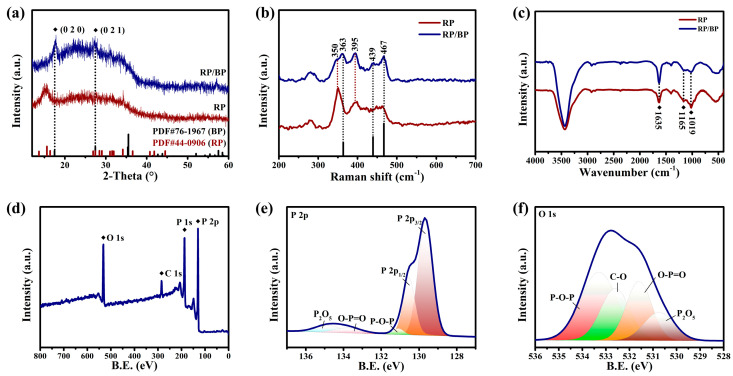
Spectroscopic characterization of the RP/BP heterojunction and bulk RP. (**a**) XRD spectra; (**b**) Raman spectra; (**c**) FT−IR spectra; (**d**) XPS survey spectrum; High-resolution XPS spectra of (**e**) P 2p and (**f**) O 1s.

**Figure 4:**
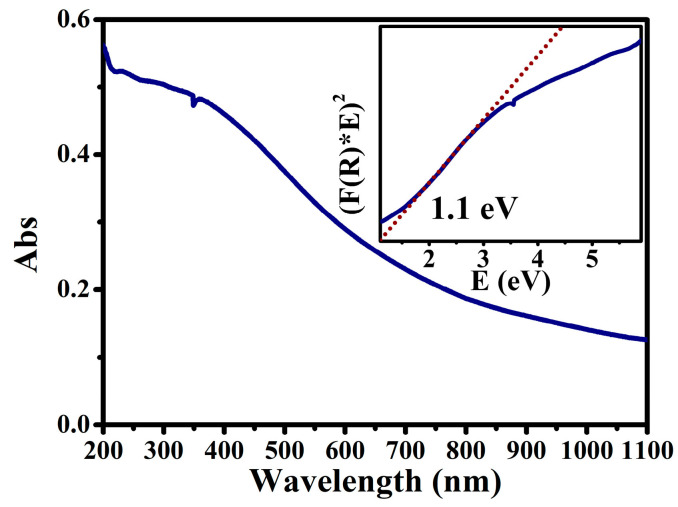
UV-vis-NIR absorption spectrum of the RP/BP heterojunction with the inset showing the band gap spectrum converted from it.

The phase structure of the RP/BP heterojunction and the bulk RP is studied by X-ray diffraction (XRD) (Figure 3a). The broad diffraction peak from 10 to 40° suggests a disordered structure of RP. The sharp diffraction peak of bulk RP at 15° is ascribed to a medium-range ordered structure. The peak positions match well with the PDF card (ID: 44-0906) of RP. The RP/BP heterojunction does not have this peak at 15°, as the thickness and size of the RP/BP heterojunction are reduced by ultrasonic treatment. The peaks at 17.6° and 27.4° correspond to (0 2 0) and (0 2 1) planes of the BP phase, respectively. These peak positions match well with the PDF card (ID: 76-1967) of BP. The molecular vibrations of the RP/BP heterojunction are characterized by Raman spectroscopy (Figure 3b). The characteristic peaks of the RP/BP heterojunction at 350 and 395 cm^−1^ are attributed to B1 fundamental mode and A1 symmetric stretching modes of RP, respectively [33]. The characteristic peaks of the RP/BP heterojunction at 363, 439, and 467 cm^−1^ belong to A_g_^1^ (out-of-plane mode), B_g_^2^ and A_g_^2^ (in-plane mode) of BP, respectively [34]. The functional groups of the RP/BP heterojunction are characterized by Fourier transform infrared (FT−IR) spectroscopy (Figure 3c). The peaks located at 1635, 1165, and 1019 cm^−1^ are assigned to P=O, P-O, and P-P-O stretching vibrations, respectively [35]. Due to the weak infrared activity and low content of BP, the FT−IR result only shows the peaks of the main RP. The peaks from left to right in X-ray photoelectron spectroscopy (XPS) survey spectra are O 1s, C 1s, P 1s, and P 2p of the RP/BP heterojunction (Figure 3d). The C element is related to the adsorbed carbon dioxide. As shown in Figure 3e, the high-resolution XPS spectrum of the P element is divided into zero-valence-state main body (P 1s and P 2p) and high-valence-state surface oxides (P_2_O_5_, O-P=O, and P-O-P). As the binding energy moves towards higher fields, the oxidation state of P also increases continuously. The high-resolution XPS spectrum of the O element indicates that the surface oxides in the RP/BP heterojunction contain P and C (Figure 3f). The ground state absorption properties of the RP/BP heterojunction are characterized by the ultraviolet-visible-near infrared (UV-vis-NIR) absorption spectrum (Figure 4). This material has a wide absorption range in the visible region. According to the Kubelka–Munk theory [36] and Tauc plot equation [37], the bandgap of the RP/BP heterojunction is confirmed to be ~1.1 eV.

**Figure 5:**
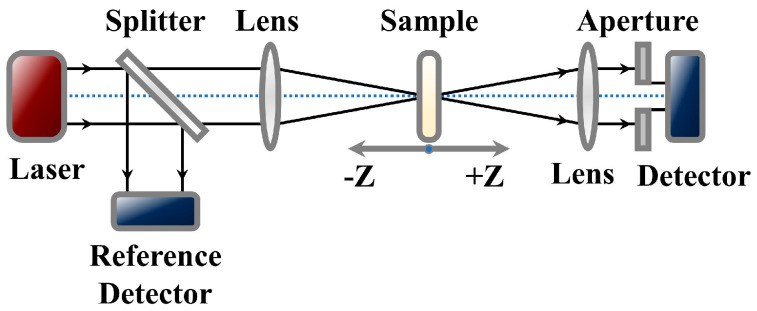
Schematic illustration of optical setup for Z−scan.

**Figure 6:**
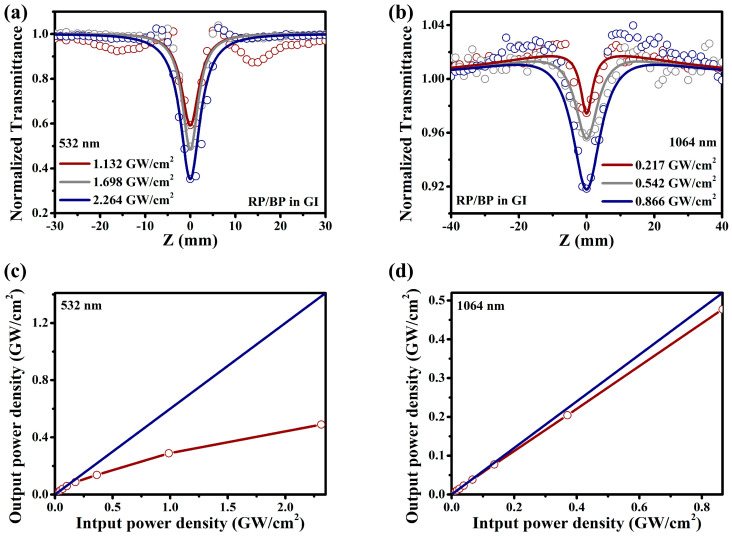
Nonlinear absorption performance of the RP/BP heterojunction in GI. Open aperture Z−scan spectra at different waist power densities under (**a**) 532 nm and (**b**) 1064 nm; Laser transmittance spectra converted from open aperture Z−scan spectra under (**c**) 532 nm (2.264 GW/cm^2^) and (**d**) 1064 nm (0.866 GW/cm^2^).

The NLO performance is characterized by the optical setup in Figure 5. The laser and optical instrument parameters can be found in Section 3.4 on characterization. A laser beam is incident on the beam splitter at an angle of 45°. One beam with half the energy of the incident laser is reflected and perpendicular to the original beam. It is collected by a reference detector to reduce laser noise. The other beam with half the energy of the incident laser can pass through the splitter and exit parallel to the original laser beam. It is focused through a convex lens. As the sample approaches the position of Z = 0, the laser power density continuously increases. By detecting the input and output laser power densities, the changes in transmittance of the sample are calculated and recorded on the Z−scan curves. The solvent GI is used to adjust the transmittance of the dispersed sample to 60%. The RP/BP heterojunction is uniformly dispersed into GI through ultrasonic treatment. At 532 and 1064 nm, the curves of the RP/BP heterojunction are both reverse saturable absorption signals with downward orientations (Figure 6a,b). Additionally, the modulation depth (defined as the difference between the maximum and minimum normalized transmittances) increases with incident laser power density. At 532 nm, the modulation depths are 0.41, 0.51, and 0.65 for power densities of 1.132, 1.698, and 2.264 GW/cm^2^, respectively (Figure 6a). At 1064 nm, the modulation depths are 0.03, 0.05, and 0.08 for power densities of 0.217, 0.542, and 0.866 GW/cm^2^, respectively (Figure 6b). The overall absorption of the dispersion sample of the RP/BP heterojunction can be expressed as:

$$\alpha \left(I\right)=\frac{\alpha_{0}}{1+I/I_{s}}+\beta \cdot I$$

where $\alpha \left(I\right)$ is the overall absorption coefficient, $\alpha_{0}$ is the linear absorption coefficient, $I_{s}$ is the saturable absorption intensity, β is the nonlinear absorption coefficient, and I is the incident laser intensity. The Z−scan curves can be non-linear fitted by using the following expression:

$$T=[1-\alpha \left(I\right)\cdot l]/(1-\alpha_{0}\cdot l)$$

$$I=\frac{I_{0}}{1+z^{2}/z_{0}^{2}}$$

where l is the sample thickness, $I_{0}$ is the light intensity at the waist, and $z_{0}$ is the Rayleigh length of the focused beams. The imaginary parts of third-order NLO susceptibilities are calculated through the following expression:

Imχ3(esu)=c2·n02240·π2·ωβ(m/W)

where Imχ3 is the imaginary part of the third order optical susceptibility, c is the velocity of light, $n_{0}$ is the linear refraction, and ω is the angular frequency of light.

The nonlinear absorption coefficients and the imaginary parts of third-order NLO susceptibilities are calculated through fitted values. At 532 nm, the nonlinear absorption coefficient of the RP/BP heterojunction is 3.47 cm/GW under 2.264 GW/cm^2^. The imaginary part of third-order NLO susceptibility is 2.34 × 10^−11^ esu under 2.264 GW/cm^2^. At 1064 nm, the nonlinear absorption coefficient of the RP/BP heterojunction is 0.72 cm/GW under 0.866 GW/cm^2^. The imaginary part of third-order NLO susceptibility is 9.72 × 10^−12^ esu under 0.866 GW/cm^2^.

Figure 6c,d shows laser transmittance spectra converted from open aperture Z−scan spectra (Figure 6a,b). This conversion calculates the input power density based on the position Z from the focus. The obtained input power density is used as the independent variable. The input power density is multiplied by the transmittance to obtain the output power density. The obtained output power density is used as the dependent variable. By converting the Z−scan curves into laser transmittance curves, it is found that their transmittances at 532 and 1064 nm decrease with the increase of laser energy. The slopes of the blue lines are both 0.6 at 532 and 1064 nm, indicating that the linear transmittance of the dispersion sample is 60%. The conversion equations between Z−scan spectrum and laser transmittance spectrum are as follows:

$$\omega^{2}\left(z\right)=\omega_{0}^{2}\cdot \left(1+\frac{z^{2}}{z_{0}^{2}}\right)$$

$$P\left(z\right)=\frac{E}{\pi \cdot \omega^{2}\left(z\right)\cdot \tau }$$

where $\omega \left(z\right)$ is the radius of the laser beam, $\omega_{0}$ is the waist radius (in this work, $\omega_{0}=13.55$ and 27.11 μm for 532 and 1064 nm, respectively), z is the distance from the waist, $z_{0}$ is the Rayleigh length, $P\left(z\right)$ is the laser power density, E is the laser energy, and τ is the pulse duration (in this study, τ = 6–7 and 8–10 ns for 532 and 1064 nm, respectively).

**Figure 7:**
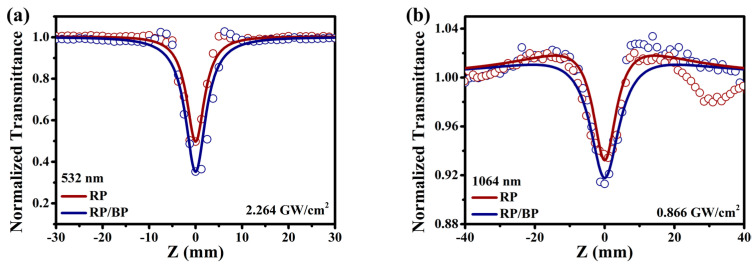
Comparison of nonlinear absorption performance between the RP/BP heterojunction and the RP in GI. Open aperture Z−scan spectra under (**a**) 532 nm and (**b**) 1064 nm.

**Figure 8:**
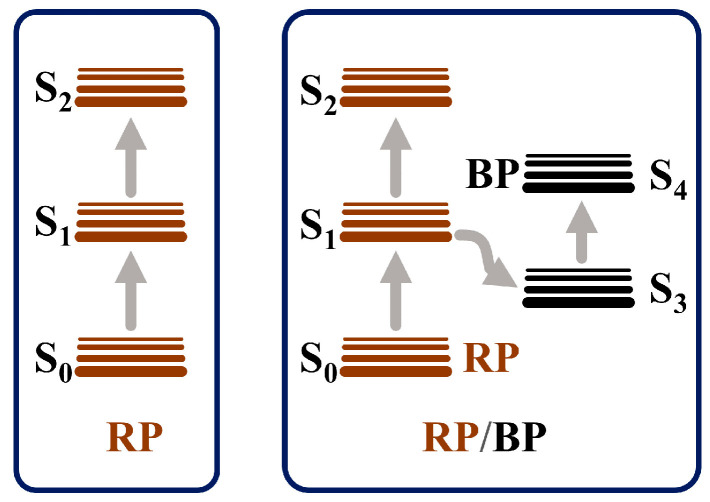
Schematic illustration of energy-level model for the RP and the RP/BP heterojunction.

The RP/BP heterojunction exhibits better NLO performance than the bulk RP, whether at 532 or 1064 nm. At the same laser power density (2.264 GW/cm^2^), the modulation depth of the RP/BP heterojunction at 532 nm is 0.65, while the modulation depth of the bulk RP is 0.50 (Figure 7a). At the same laser power density (0.866 GW/cm^2^), the modulation depth of the RP/BP heterojunction at 1064 nm is 0.08, while the modulation depth of the bulk RP is 0.07 (Figure 7b). Compared with the bulk RP, the modulation depths of the RP/BP heterojunction increase by 28.8% and 32.6% at 532 and 1064 nm, respectively. The reverse saturable absorption of the RP without the introduction of BP is weaker than that of the RP/BP heterojunction with the introduction of BP. As shown in Figure 8, the energy level of the bulk RP follows a three-level model. It is determined that the reverse saturable absorption of bulk RP is not two-photon absorption. As shown in Figure 7b, if it is two-photon absorption, a slightly upward signal cannot occur. Only when the excited state absorption cross-section is larger than that of the ground state absorption will the reverse saturable absorption of the bulk RP arise. The energy level of the RP/BP heterojunction follows a five-level model [26]. In contrast to the bulk RP, the charge transfer occurs in the RP/BP heterojunction due to the introduction of the BP phase [26]. Because of the larger absorption cross-section of BP and longer carrier relaxation time, the excited state absorption of the RP/BP heterojunction is enhanced. Therefore, its reverse saturable absorption performance is stronger.

**Figure 9:**
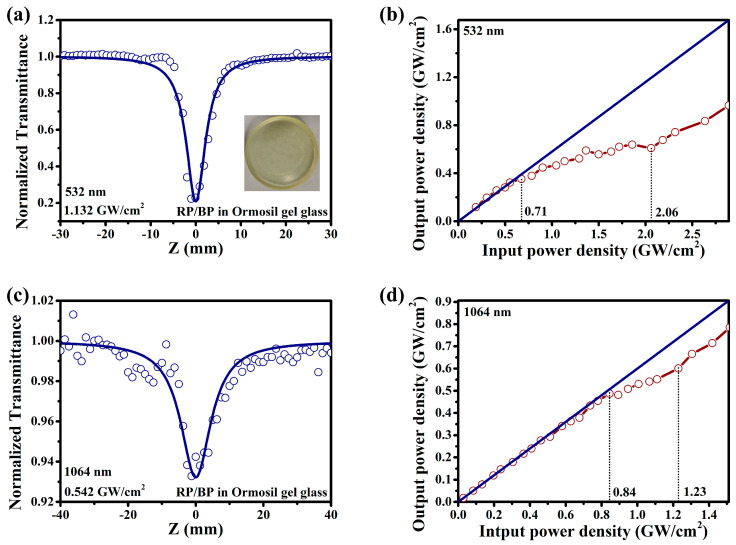
OL performance of the RP/BP heterojunction doped ormosil gel glass. Open aperture Z−scan spectra under (**a**) 532 nm (with the optical image of the RP/BP heterojunction doped silicone gel glass in the inset) and (**c**) 1064 nm; Laser transmittance spectra under (**b**) 532 nm and (**d**) 1064 nm.

The RP/BP heterojunction is doped in ormosil gel glass for practical application in OL. As shown in the inset of Figure 9a, the RP/BP heterojunction doped silicone gel glass exhibits a transparent yellow color. As shown in Figure 9a,c, the RP/BP heterojunction doped silicone gel glass has excellent broadband reverse saturable absorption performance. At 532 nm, the modulation depth of the RP/BP heterojunction doped ormosil gel glass reaches 0.78 at 1.132 GW/cm^2^ (Figure 9a). At 1064 nm, the modulation depth of the RP/BP heterojunction doped ormosil gel glass is 0.068 at 0.542 GW/cm^2^ (Figure 9c). By fitting and calculating, the nonlinear absorption coefficients of the RP/BP heterojunction doped ormosil gel glass at 532 and 1064 nm are 4.23 and 0.75 cm/GW, respectively. The imaginary parts of third-order NLO susceptibilities of the RP/BP heterojunction doped ormosil gel glass at 532 and 1064 nm are 2.85 × 10^−11^ and 1.01 × 10^−11^ esu, respectively. The laser transmittance of the RP/BP heterojunction doped ormosil gel glass at different laser power densities is characterized in Figure 9b,d. These spectra are obtained by placing the sample at the waist of the laser beam of the optical system and recording the input and output power densities. In instances where laser power density is low, the majority of data points tend to cluster around the blue straight line (Figure 9b). This means that the absorption of the RP/BP heterojunction in ormosil gel glass is linear at low laser power density. In instances where laser power density is higher, the transmittance of the sample demonstrates a continuous decrease as the laser power density increases. At 532 nm, the initial limiting threshold of the RP/BP heterojunction doped ormosil gel glass is 0.71 GW/cm^2^. The laser damage threshold is 2.06 GW/cm^2^. After this point, the transmittance of the Z−scan curve starts to increase again. Similarly, at 1064 nm, the sample exhibits nonlinear absorption as the laser power density increases (Figure 9d). At 1064 nm, the initial limiting threshold of the RP/BP heterojunction doped ormosil gel glass is 0.84 GW/cm^2^. The laser damage threshold is 1.23 GW/cm^2^. At both wavelengths, this device shows capacity to regulate the laser power density within a certain range, demonstrating the potential value of the RP/BP heterojunction doped ormosil gel glass for OL applications.

## 3. Materials and Methods

### 3.1. Materials

Bulk red phosphorus and ethylenediamine were purchased from Aladdin Biochemical Technology Co., Ltd (Shanghai, China). Black phosphorus was purchased from Zhongke Experimental Materials Co., Ltd (Guangzhou, China). Methyltriethoxysilane and acetic acid were purchased from InnoChem Science & Technology Co., Ltd (Beijing, China). All chemicals were analytical grade.

### 3.2. Preparation of RP/BP Heterojunction

Preparation of the RP/BP heterojunction followed the method described previously [26]. Firstly, red phosphorus was purified by hydrothermal treatment to remove the oxide layer. Next, bulk red phosphorus (3 g) was dispersed in deionized water (80 mL). Then, the mixture was transferred to a Teflon-lined stainless autoclave (100 mL) and maintained at 200 °C for 12 h. Secondly, the purified red phosphorus was dispersed in ethylenediamine (30 mL). The mixture was then transferred to a Teflon-lined stainless autoclave (50 mL) and maintained at 140 °C for 12 h. After cooling down to room temperature, black precipitate was collected by centrifugation and washing with deionized water and ethanol. The precipitate was placed in an oven (50 °C) for drying. The precipitate (150 mg) was added into deionized water (200 mL). Under ice bath conditions, the dispersion was treated with ultrasound for 1 h to exfoliate layers. Finally, the RP/BP heterojunction was obtained by centrifugation, washing, and drying as described above.

### 3.3. Preparation of RP/BP Heterojunction Doped Ormosil Gel Glass

Firstly, methyltriethoxysilane (21.25 mL), deionized water (6.75 mL), ethanol (75 mL), and acetic acid (0.4 mL) were mixed in a beaker (200 mL) and stirred for 24 h. Then, a portion of the solvents (~50 mL) in the mixture was removed by a vacuum distillation device. The remaining mixture was stirred continuously for 6 d. The RP/BP heterojunction (0.005 g) was fully dispersed in the as-prepared prepolymer solution (11.03 mL). Subsequently, the dispersion was transferred to a mold and dried at room temperature for about 6 d. After demolding, the RP/BP heterojunction doped ormosil gel glass was obtained.

### 3.4. Characterization

The TEM images were characterized by a Hitachi HT7700 (Hitachi Energy, Tokyo, Japan). The HRTEM and EDS elemental mappings were characterized by a FEI Tecnai G2 F20 (DEI, Hillsboro, OR, USA). The AFM image was characterized by a Bruker Multimode 8 (Bruker, Berlin, Germany). The SEM image was characterized by a Hitachi S-4800 (Japan). The XRD spectra were characterized by a Bruker D8 focus (Germany). The Raman spectra were characterized by a Renishaw inVia-Qontor (Renishawm London, UK). The FT−IR spectra were characterized by a Varian Excalibur 3100 (Varian, Washington, DC, USA). The XPS spectra were characterized by a ThermoFisher Scientific Escalib 250Xi (Thermo Fisher, London, UK). The UV-vis-NIR absorption spectrum was characterized by a Varian Cary 7000 (United States of America). The Z−scan spectra and laser transmittance spectra were characterized by a Spectra Physics Premiscan/240/MB-ULD (Spectra Physics, Milpitas, CA, USA). An Nd: YAG laser was used in the system with pulse durations of 6–7 ns (for 532 nm) and 8–10 ns (for 1064 nm) and a repetition rate of 10 Hz. The focal length of the convex lens in the optical path was 200 mm. The Rayleigh lengths of the focused beams were 1.08 mm (for 532 nm) and 2.17 mm (for 1064 nm). The detector used was a dual channel energy meter. 

## 4. Conclusions

By using bulk RP as the precursor, an RP/BP heterojunction (with a thickness of ~1.5 nm) has been successfully prepared by solvothermal and ultrasonic methods. The solvent ethylenediamine plays an important role in inducing the phase transition. At 532 and 1064 nm, the reverse saturable absorption of the RP/BP heterojunction is observed in GI. The nonlinear absorption coefficient of the RP/BP heterojunction in GI is 3.47 (532 nm, 2.264 GW/cm^2^) and 0.72 cm/GW (1064 nm, 0.866 GW/cm^2^), respectively. The imaginary parts of third-order NLO susceptibility of the RP/BP heterojunction in GI are 2.34 × 10^−11^ (532 nm, 2.264 GW/cm^2^) and 9.72 × 10^−12^ esu (1064 nm, 0.866 GW/cm^2^), respectively. Due to charge transfer, the RP/BP heterojunction exhibits obvious enhancement in nonlinear absorption compared to RP (by 28.8% and 32.6% at 532 and 1064 nm, respectively). For practical application in a device, an RP/BP heterojunction doped ormosil gel glass has been prepared, which has excellent broadband NLO performance. The imaginary part of third-order NLO susceptibility of this device reaches 2.85 × 10^−11^ esu at 532 nm. The initial limiting threshold of this device is 0.71 GW/cm^2^ at 532 nm. This device can limit the output laser power density within a certain range at 532 and 1064 nm, demonstrating a great potential for application in OL or many other fields.

## Data Availability

The dataset is available on request from the authors.
